# Effects of inspiratory muscle training on lung function parameter in swimmers: a systematic review and meta-analysis

**DOI:** 10.3389/fspor.2024.1429902

**Published:** 2024-09-16

**Authors:** Nathali Carvajal-Tello, José Guillermo Ortega, Andrés Fabricio Caballero-Lozada, María Juliana Devia-Quiñonez, Isabella González-Calzada, Daniela Rojas-Hernández, Alejandro Segura-Ordoñez

**Affiliations:** ^1^ Grupo de Investigación Salud y Movimiento, Faculty of Health, Universidad Santiago de Cali, Cali, Colombia; ^2^ Grupo de Investigación de Ciencias Básicas y Clínicas de la Salud, Department of Basic Health Sciences, Pontificia Universidad Javeriana, Cali, Colombia; ^3^ Grupo de Investigación en Anestesia INVANES, Department of Anesthesiology and Resuscitation, Universidad del Valle, Cali, Colombia; ^4^ Department of Anesthesiology, Hospital Universitario del Valle, Cali, Colombia; ^5^ Intensive Care Unit, Fundación Hospital San José de Buga, Buga, Colombia

**Keywords:** athletes, swimming, muscle strength, respiratory muscles, sports performance

## Abstract

**Background:**

This systematic review and meta-analysis aimed to assess the impact of inspiratory muscle training (IMT) on lung function parameters (MIP, MEP, FEV1, and FVC) between both elite and non-elite swimmers.

**Methods:**

We searched for controlled clinical trials (CCT) and prospective longitudinal studies (PLS) in elite and non-elite swimmers following an inspiratory muscle training (IMT) protocol with a standardized device, published between 2012 and 2023. The databases used in the search were PubMed, Science Direct, Scopus, Springer, Cochrane Central Register of Controlled Trials, and Google Scholar. The primary outcome assessed was the impact of IMT on lung function parameters, including MIP, MEP, FEV1, and FVC.

**Results:**

We selected 13 articles involving 277 subjects aged 11–21 years, with 61.4% being male, and 84.6% being elite swimmers. The most commonly used IMT device was the PowerBreathe®, prescribed for 3–12 weeks, 1–2 sessions per day, 3–6 times per week, with 30 repetitions, starting at 50% of MIP and progressing up to 80%. The meta-analysis showed that IMT was associated with a higher MIP (MD = 29.35 cmH2O, 95% CI: 13.04–45.65 cmH2O, *p* < 0.01) without affecting FEV1 and FVC.

**Conclusion:**

The swimmers that used IMT improved muscle strength, specifically MIP, without changes in MEP, FEV1, and FVC.

## 1 Introduction

Swimming is an activity performed for recreational or competitive purposes that involves exercising while submerged in water. It imposes significant demands on the respiratory muscles due to the higher hydrostatic pressure surrounding the thoracic cavity. This leads to an increase in the speed of inspiratory muscle contraction and tidal volume (1, 2). The workload on respiratory muscles during swimming can lead to fatigue and dyspnea. This is primarily due to these muscles having to overcome the hydrostatic pressure which limits the thoracic expansion, ultimately reducing performance (3, 4). During vigorous exercise, the demands on the respiratory system to function properly rise significantly (5). The respiratory system acts as one of the main metabolic buffers by increasing the strength of the respiratory muscles to ensure ventilation and maintain normal acid-base balance in the blood (6). The efficiency of the respiratory muscles is crucial; if they are inefficient, it could lead to a hypercapnic state where working muscles tire faster because the removal of metabolites cannot keep up with the production of CO2 (1). The high levels of sustained work required from the respiratory muscles during intense exercise can lead to respiratory muscle fatigue. However, having respiratory muscles with enhanced contractile responsiveness and increased force generation capacity could result in better fatigue tolerance and a reduced perception of breathlessness during exercise (7).

Hence, improving lung function and respiratory muscle strength has been recommended as a potential strategy to enhance physical performance in swimming (8). One suggested strategy to increase the strength of respiratory muscles, including the diaphragm, is Inspiratory Muscle Training (IMT), which compels the inspiratory muscles to overcome increased force, thereby enhancing their overall strength by introducing resistance during inspiration (9). It is well known that when postural demands increase, the activation of the diaphragm increases (10). Diaphragmatic and abdominal stability and contraction can increase glide performance by boosting gliding ability which plays a significant part in race performance, preserving the aerodynamic body posture, which is critical for swimmers (11).

Thus, is expected that IMT has an impact on parameters of pulmonary function in swimmers, including maximum inspiratory pressure (MIP), which represents the maximal force generated by the inspiratory muscles against resistance (12), reflecting their strength and endurance; the maximum expiratory pressure (MEP), an indicator of the maximal force exerted during expiration (13); the forced expiratory volume in 1 s (FEV1) which is the parameter of the volume of air forcefully exhaled within the first second of forced expiration (14); and forced vital capacity (FVC), which encompasses the total volume of air forcibly exhaled after maximum inhalation (15).

However, there is currently no consensus on the effects of Inspiratory Muscle Training (IMT) on lung function in swimmers. Therefore, knowing the impact of this type of training on physiological lung parameters is crucial to support the practice and prescription of IMT in swimming training programs. For this reason, this systematic review and meta-analysis aimed to evaluate the effect of IMT on lung function parameters (MIP, MEP, FEV1, and FVC) in both elite and non-elite swimmers.

## 2 Materials and methods

### 2.1 Literature search

We included data from CCT and PLS published in the period 2012–2023, the search was carried out in the electronic databases: PubMed, Science Direct, Scopus, Springer, Cochrane Central Register of Controlled Trials, and Google Scholar, between 01/11/2022 and 31/05/2023, without language restriction. The study complies with the preferred reporting items for systematic reviews and meta-analyses (PRISMA) (16). Mesh terms with combinations of boolean operators according to the database were used to search: (athletes OR professional athlete OR professional athletes OR athletic performance) AND (water sport OR swimming OR diving) AND (respiratory muscle OR diaphragm OR intercostal muscles OR ventilatory muscle OR muscles) AND (Respiratory Muscle Training OR muscle training, respiratory OR training OR resistance).

### 2.2 Inclusion criteria and selection process

After removing duplicated reports, two researchers independently assessed the titles and abstracts for eligibility criteria. Any disagreements were resolved by a third researcher. We included studies with elite or non-elite swimmers where IMT was performed using standardized devices such as Threshold IMT®, Threshold PEP®, and PowerBreathe®. We excluded studies that included subjects diagnosed with pulmonary, cardiac, or musculoskeletal disease, chronic or acute illness, and cognitive disorders. Additionally, we excluded systematic reviews, case series, and case reports.

### 2.3 Methodological quality

The quality of the CCT was evaluated using the PEDro scale, which consists of 11 items. A score of 1 is given if the item complies and 0 if it does not comply. The methodological quality is classified as low, intermediate or high based on the sum of the score. The aspects considered include an adequate control group, blinding, and randomization (17). The Minors scale was used to assess PLS. This scale consists of 12 items, with a score of 0 for not reported, 1 for reported but inadequate, and 2 for reported and adequate. The scale considers aspects such as objectives, prospective data collection, blinding, sample size calculation, and adequate statistical analysis, the ideal score for non-comparative studies is 16, and for comparative studies, it is 24 (18).

### 2.4 Data extraction and analysis

Two authors independently extracted data from eligible studies. The data extracted were recorded on a standardized data collection form. We included bibliographic characteristics such as authors, title, database, journal, type of study, country, continent, language, year and objective. Participant characteristics, such as sex, age, height, weight, and competitive level, were also collected. Intervention characteristics, such as the type of training, starting intensity, intensity progression, frequency per week, session, and intervention duration, were documented. The evaluation of respiratory muscle training on performance included parameters such as Maximum Inspiratory Pressure (MIP), Maximum Expiratory Pressure (MEP), Forced Expiratory Volume in the first second (FEV1), and Forced Vital Capacity (FVC). Disagreements were resolved by consensus.

### 2.5 Statistical analysis

A qualitative summary was carried out according to study design, characteristics, sample characteristics, number of subjects, type of instruments used to measure respiratory muscle training (Threshold®, PowerBreathe®), swimming performance (maximum apnea test, 50, 100, 200, 200, 3000-m pool), dyspnea (Borg scale), and lung capacity and, volumes (spirometry).

The primary outcomes for the meta-analysis, were the mean differences (MD) for MIP, FEV, and FVC. Quantitative analyses were only conducted if comparable outcome data from four or more studies were available. In cases where study data were only available from figures, we extracted the data using the validated software Plot Digitizer (19). When baseline and final values were given, we computed changes from the baseline. Missing standard deviation (SD) values were imputed using an imputed correlation coefficient (20). When a study presents multiple interventions with IMT and a unique control group, the groups IMT were combined into a single group (21). For quantitative synthesis, we used a random effects model with restricted maximum likelihood to estimate between-study variability (*τ*2) and Knapp-Hartung adjustment was used for small numbers of studies with considerable heterogeneity (22, 23). The *I*2 index was used to determine the heterogeneity, an *I*2 ≥ 75% indicated high variation. For all MDs, the reference group was the control group. The publication bias for a few studies was examined qualitatively via visual inspection of the Doi plot, and quantitatively using the Luis Furuya-Kanamori (LFK) index. LFK indices <1, between 1 and 2, and >2, represent no, minor, and major asymmetry, respectively (24). To evaluate the robustness of the results, we performed a sensitivity analysis by excluded one study at a time (Leave-one-out analysis). Additionally, subgroup analysis was conducted according to the type of control group. For outcomes with significant effects we performed a meta-regression, to know if some factors (age, outcome at baseline, duration of intervention) influenced the effects. The results were considered statistically significant if *p* ≤ 0.05. The statistical analyses were conducted with the statistical software R (version 4.0.3), and the packages meta (Version 6.5.0) and metasens (Version 1.5.2) (25, 26).

## 3 Results

**Figure 1:**
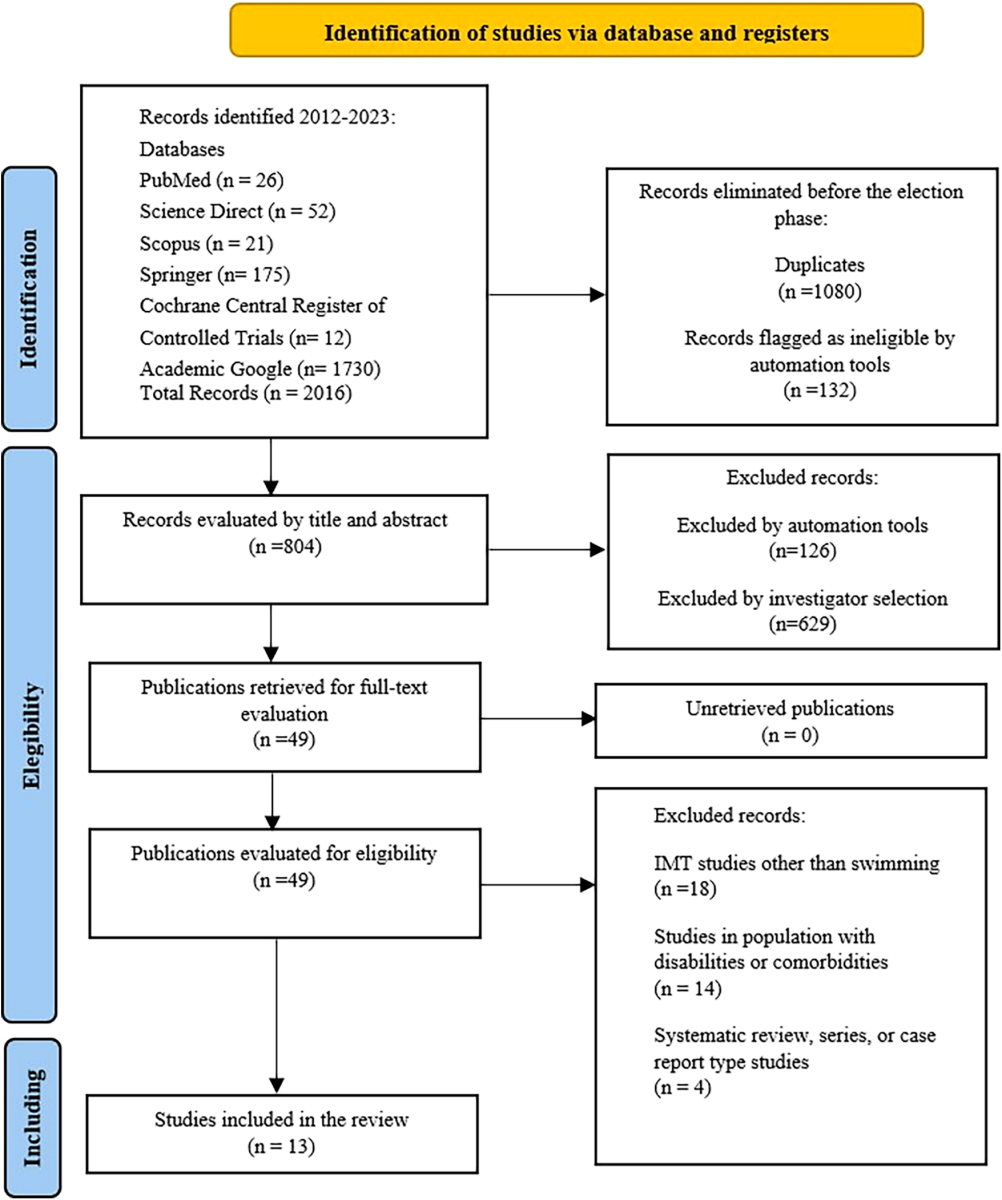
Prisma flow diagram.

In this systematic search, we obtained a total of 2016 reports. After eliminating 1,212 duplicates, 804 articles were assessed based on titles and abstracts, of which 755 were excluded. Subsequently, 49 full-text articles were reviewed, and 36 studies were excluded either because they did not meet the inclusion criteria or because they had one or more exclusion criteria. A total of 13 articles were selected for the meta-analysis (Figure 1).

### 3.1 Characteristics of the studies

Of the 13 studies selected, 54% (*n* = 7) were found in Pubmed and 42% (*n* = 4) in Google Scholar. According to design, 11 studies were controlled clinical trials (CCT) and 2 studies were prospective longitudinal studies (PLS). Considering a geographical region, 54% (*n* = 7) were from Europe, 23% (*n* = 3) from Asia, 23% (*n* = 3) from America, 92% (*n* = 12) of the articles were written in the English language (Table 1).

**Table 1:** Characteristics of the studies included.

#	Authors	Title	Database	Journal	Type of study	Country	Continent	Language	Year	Objective
1	Vašíčková et al. (27)	The effect of respiratory muscle training on the performance of finswimmers	Pubmed	Journal of Sports Science and Medicine	CCT	Czech Republic	Europe	English	2017	To evaluate the response to IMT in fin swimmers on their maximal apnea performance
2	Kapus (28)	Effects of IMT on inspiratory muscle strength and sprint swimming performance in young male and female swimmers	Academic Google	Kinesiologia Slovenica	CCT	Slovenia	Europe	English	2013	To explore the effects of IMT on inspiratory muscle strength measured on land in an upright position and sprint swimming performance (50 m and 100 m) in different swimming techniques (crawl, breaststroke and butterfly) in young swimmers
3	Yañez-Sepulveda et al. (29)	IMT improves swimming performance in young competitive male sprint swimmers	Pubmed	The Journal of sports medicine and physical fitness	CCT	Chile	America	English	2021	To observe the effect of IMT on swimming performance and its relationship with inspiratory force and lung function
4	Lomax et al. (30)	Impact of weekly swim training distance on the ergogenicity of IMT in well-trained young swimmers	Pubmed	J Strength Cond Res	CCT	London	Europe	English	2019	To examine the impact of weekly swim training distance on IMT ergogenicity
5	Ando et al. (31)	Effect of inspiratory resistance training on diaphragm shear modulus and accessory inspiration	Academic Google	Journal of the Japanese Society of Respiratory Care and Rehabilitation	CCT	Japan	Asia	English	2020	To elucidate changes in the diaphragm and inspiratory accessory muscles (sternocleidomastoid muscle and intercostal muscle) as they function after a 29-week training program
6	Cunha et al. (32)	The effect of IMT on swimming performance, inspiratory muscle strength, lung function and perceived dyspnea in elite swimmers: a randomized controlled trial.	Pubmed	Porto Biomedical Journal	CCT	Portugal	Europe	English	2019	To evaluate the effect of 12-week IMT on swimming performance, inspiratory muscle strength, lung function, and perceived dyspnea in elite swimmers
7	Ohya et al. (33)	Effect of moderate to high intensity IMT on mouth-measured PIM and swimming performance in highly trained competitive swimmers	Pubmed	Human Kinetic Journal	CCT	Tokio	Asia	English	2021	To identify the effect of high-intensity IMT for 6 weeks on MIP and swimming performance in highly trained competitive swimmers
8	Wilson et al. (34)	Specific respiratory muscle warm-up and elite swimming performance	Pubmed	British Journal of Sport Medicine	PLS	Nottingham	Europe	English	2014	To determine the influence of inspiratory muscle exercise as a warm-up for the respiratory muscles
9	Mackała et al. (35)	Effects of individualized training and IMT on lung function in collegiate swimmers: an experimental study	Academic Google	Physical Education Theory and Methodology	CCT	Malasia	Asia	English	2022	To examine the effect of individualized training and respiratory muscle training on lung function among collegiate swimmers
10	Shei et al. (36)	Effect of flow-resistant inspiratory loading on lung and respiratory muscle function in sub-elite swimmers.	Scopus	Journal of Sports Medicine and Physical Fitness	CCT	Indiana	America	English	2016	To evaluate the effects of a 12-week swimming and IMT training program on respiratory muscles and lung function in competitively trained sub-elite swimmers
11	Troncoso et al. (37)	Effect of IMT on swim time and lung function in young high performance swimmers	Academic Google	Revista Chilena de Rehabilitación y Actividad Física	PLS	Chile	America	Spanish	2021	To examine the effect of IMT on swim time and lung function in young high performance swimmers
12	Bağıran et al. (38)	The effect of IMT on aerobic power and respiratory parameters in swimmers	Academic Google	International Journal of Sport Exercise and Training Sciences—IJSETS	CCT	Turkey	Europe	English	2019	To investigate the effect of 6 weeks of IMT on VO2 max and respiratory parameters in swimmers
13	Gómez-Albareda et al. (39)	IMT improves PIM without increasing performance in elite swimmers	Pubmed	International Journal of Sports Physiology and Performance	CCT	Spain	Europe	English	2023	To analyze the effect of IMT on PIM and performance of elite swimmers

IMT, inspiratory muscle training; VO2 max, maximal oxygen consumption; CCT, controlled clinical trials; PLS, prospective longitudinal studies; PIM, peak inspiratory pressure.

The study included a total of 277 subjects aged between 11 and 21 years, with 61.4% (*n* = 170) being male. Four studies exclusively included male subjects, while 9 studies included both sexes. The height of the subjects ranged from 153 ± 0.18 to 179.42 ± 0.74, and their weight ranged from 47.4 ± 10.5 to 75.2 ± 9.05 kg (Table 2). The participants were predominantly elite swimmers, comprising 84.6% (*n* = 11), with 1–10 years of experience and a training time of 6–35 h per week. Two studies (15.4%; *n* = 2) were conducted on non-elite swimmers who trained three times per week.

**Table 2:** Characteristics of the participants of the studies included.

First author, year	*n*	F/M	EG/CG	Age (years)	Height (cm)	Weight (kg)	CL	Inclusion criteria	Exclusion criteria
Vašíčková et al. (27)	20	N/S	EG = 12	EG = 12.0 ± 1.7	EG = 158 ± 0.11	EG = 47.4 ± 10.5	Elite	Participate in fin swimming training at a swim club twice a week for at least 2 years	Chronic or acute illness
			CG = 8	CG = 11.5 ± 2.4	CG = 153 ± 0.18	CG = 49.6 ± 17.0			
Kapus (28)	12	F = 7	EG = 7	EG = 14 ± 1	EG = 169 ± 4	EG = 57 ± 6	Elite	Competitive swimmers for at least 6 years and recruited from two swimming clubs, mostly national level sprint and middle distance specialists	Respiratory disease
		M = 5	CG = 5	CG = 14 ± 1	CG = 172 ± 10	CG = 59 ± 12			
Yañez-Sepulveda et al. (29)	15	M = 15	EG = 9	EG = 15.1 ± 1.1	EG: 174 ± 5.16	EG = 62.8 ± 7.31	Elite	Have a minimum of 3 years of systematic training with more than 10 h of training per week and no previous experience with IMT	Restrictions or obstructive respiratory disorders, those who did not perform more than 90% of the IMT sessions, or those who had suffered a serious injury in the previous 6 months
			CG = 6	CG = 14.7 ± 1.09	CG = 173 ± 8.14	CG = 61.3 ± 6.57			
Lomax et al. (30)	33	F = 15	EG = 17	LOWIMT = 16 ± 1	LOWIMT = 175 ± 0.11	LOWIMT = 65.2 ± 8.3	Elite	Years of competitive experience: 3–5, frequency of weekly sessions: 6–9, duration in hours of weekly training: 10.5–19, weekly training distance in km: 15–56	If asthma is present, no medication is required during the study
			LOWIMT = 9	HIGHIMT = 16 ± 3	HIGHIMT = 176 ± 0.12	HIGHIMT = 65.9 ± 13.7			
			HIGHIMT = 8						
		M = 18	CG = 16						
			LOWIMT = 9						
			HIGHIMT = 7						
Ando et al. (31)	19	M = 19	EG = 10	EG = 19.3 ± 0.1	EG = 173.5 ± 0.6	EG = 68.9 ± 0.8	Elite	Elite swimmers	N/S
			CG = 9	CG = 19.3 ± 0.1	CG = 174.4 ± 0.5	CG = 69.0 ± 0.9			
Cunha et al. (32)	32	F = 22	EG = 17	EG = 15[14;16]	EG = 171 (9)	EG = 60.9 (9.3)			
		M = 10	CG = 15	CG = 14 [13;16]	CG = 164 (11)	CG = 56.7 (11.6)	Elite	Elite swimmers from the FC Porto main swimming team, with competitive training for a minimum period of 3 years	Diagnosed lung disease, heart disease, musculoskeletal disease, cognitive disorders, or being on a regular respiratory muscle training program
Ohya et al. (33)	30	M = 30	EG (HI) = 10	EG (HI) = 20 (1)	EG (HI) = 175.4 (4.4)	EG (AI) = 69.1 (4.6)	Elite	All participants were required to be a member of the college swim club and have participated at least once in interscholastic and intercollegiate athletic competition	N/S
			EG (MI) = 10	EG (MI) = 19 (1)	EG (MI) = 173.5 (6.4)	EG (MI) = 68.9 (7.5)			
			CG = 10	CG = 19 (1)	CG = 174.4 (4.0)	CG = 69.0 (7.8)			
Wilson et al. (34)	15	F = 6	EG = 15	21.2 ± 1.6	180.17 ± 7.84	75.2 ± 9.05	Elite	Have been selected to represent British swimming in international competitions	Not completing 100% of IMT sessions and physiological measurements
		M = 9							
Mackała et al. (35)	43	F = 21	EG EMI = 14	EG EMI = 19.50 ± 1.225					
		M = 22	EG EMI + IT = 15	EG EMI + IT = 19.60 ± 1.121	N/E	N/E	Non elite	Healthy swimmers 18-25 years of age, non-elite freestyle swimmers, swimming at least three days a week	Recent lung infections, congenital lung problems, known history of respiratory problems such as asthma, COPD, recent injuries to lower or upper limbs, BMI greater than 24 kg/m^2^, and those participating in any other research
			CG = 14	CG = 19.50 ± 1.092					
Shei et al. (36)	24	F = 12	EG = 8	19.9 ± 2.6	173 ± 3.8	67.6 ± 10.4	Non elite	Sprint swimmers (50–100 m), 14 middle distance (200–400 m) and 2 long distance (800–1500 m).	Abnormal spirometry or history of respiratory disease
		M = 12	PG = 8						
			CG = 8						
Troncoso et al. (37)	6	F = 1	EG = 6	15.7 ± 1.0	167.3 ± 5.5	60.3 ± 7.8	Elite	Age between 15 and 17 years old, belonging to the swimming team of the Maule region for one year, completing 18 h of training in 9 weekly sessions	Respiratory or musculoskeletal disease in the study period
		M = 5							
Bağıran et al. (38)	20	M = 20	EG = 10	EG = 20.05 ± 2.62	EG = 179.42 ± 0.74	EG = 71.32 ± 4.12	Elite	Male swimmers between the ages of 18 and 23. Voluntarily participate in a training program	N/S
			CG = 10	CG = 20.56 ± 1.16	CG = 178 ± 0.32	CG = 72.24 ± 5.11			
Gómez-Albareda et al. (39)	8	F = 3	EG = 4	EG = 21.0 [2.6] CG = 18.3 [1.3]	EG = 173.9 [8.8]	EG = 64.5 [6.3] CG = 66.5 [3.9]	Elite	10 years of experience in swimming training, performing more than 35 h of training per week and a weekly volume of swimming training greater than 75 km	N/S
		M = 5	CG = 4		CG = 179.4 [7.0]				

NC, competitive level; N/S, not specific; EG, experimental group; CG, control group; PG, placebo group; HI, high intensity; MI, moderate intensity; IMT, inspiratory muscle training; IT, individualized training; BMI, body mass index, LOWIMT, low distance + inspiratory muscle training; HIGHIMT, long distance + inspiratory muscle training.

### 3.2 Characteristics of interventions

The IMT training was conducted using the PowerBreathe® device in 92.3% (*n* = 12) of the studies (Table 3). The initial intensity of IMT ranged from 15% to 80% of the MIP, with a progression in training intensity and loads from 5% to 50% per week; in two studies, no progression protocol was specified (30, 31). Concerning the frequency and duration of IMT application, the majority of the reports (27–33, 37–39) implemented 2 sessions/day, 6 times/week over 4–12 weeks, with the duration of the session varying between 10 and 30 inspiratory efforts. One study reported a 15-min duration with the training device (27).

**Table 3:** Description of the interventions included in the studies.

First author, year	Training type	Start intensity	Training intensity progression	Sessions per day/times per week	Number of weeks	Session duration	EG/CG
Vašíčková et al. (27)	Threshold PEP® Threshold IMT®	EG = 30% MIP	Each week the initial resistance value was increased by 2 CmH2O. When the participants reached the maximum resistance possible on the devices, they trained with this maximum resistance on the devices for the remainder of the training period	2 s/N/S	4	EG = RMST = 10 repetitions of maximum inspiration (Threshold IMT) and 10 repetitions of maximum expiration (Threshold PEP). CG = RMET = 15 min with each device (IMT Threshold) (PEP Threshold)	EG = IMT
		CG = RST					CG = RST
Kapus (28)	POWER breathe®	EG = 50% MIP	EG = Load increase periodically once a week up to 30 manoeuvres. CG = Training loads did not change throughout the training period	2 s/N/S	6	EG = 30 dynamic inspiratory efforts 50% MIP	EG = IMT 50% MIP + RST CG = IMT 15% MIP + RST
		CG = 15% MIP				CG = 30 breaths slowly held 15% MIP	
Yañez-Sepulveda et al. (29)	POWER breathe®	EG = 50% MIP	EG = Only this group increased the initial load by 5% each week	2 s/N/S	4	EG = 30 dynamic inspiratory efforts 50% MIP	EG = IMT 50% MIP + RST
						CG = 30 slowly held breaths 15% MIP	CG = IMT 15% MIP + RST
		CG = 15% MIP					
Lomax et al. (30)	POWER breathe®	LOW IMT—HIGH IMT = 50% MIP	For both groups the same MIP intensity was maintained, the RST distance was low and high	2 s/6 t	6	LOW IMT y HIGH IMT = 30 maximum inhalations at 50% MIP	HIGH IMT = RST High distance 42–56 Km + IMT
							LOW IMT = RST Low distance 15–31 Km + IMT
Ando et al. (31)	POWER breathe®	EG = 50% MIP	The same MIP load was maintained in the EG and only the RST was performed in the CG	2 s/6 t	6	EG = RMST = 30 maximum inhalations at 50% MIP	EG = IMT + RST
		CG = RST					CG = RST
Cunha et al. (32)	POWER breathe®	EG = 50% MIP	EG = Increase the load up to 30 inspiratory maneuvers. CG = Maintain the inspiratory load throughout the intervention	2 s/5 t	12	EG = RMST = 2 cycles of 30 inspiratory efforts at an intensity of 50% MIP	EG = IMT 50% + RST
		CG = 15% MIP				CG = RMST = 2 cycles of 30 inspiratory efforts at an intensity of 15% MIP	CG = IMT 15% + RST
Ohya et al. (33)	POWER breathe®	EG (HI) = 75% MIP	EG (HI) = Week 1–2: 50% MIP, week 3–6: increases to 75% MIP EG (MI) = week 1–2: 50% MIP, week 3–6 maintains MIP 50%	2 s/6 t	6	EG = (HI) and (MI) = 30 maximum inhalations with intensity of 75% MIP (HI) and 50% (MI)	EG (HI) y (MI) = IMT, RST, GT
		EG (MI) = 50% MIP					CG = RST, GT
		CG = RST					
			CG = RST				
Wilson et al. (34)	POWER breathe®	EG = P1 = RST 2,500 m (butterfly, backstroke, breaststroke and freestyle), P2 = RST + IMT 2 series 30 repetitions 40% MIP, P3 = RST + IMT 2 series 30 repetitions 15% MIP, P4 = warm-up with IMT	Each swimmer completed all 4 protocols	1 s/N/S	4	Each swimmer was randomized to 1 of the 4 protocols each week, until completing the period of 4 consecutive weeks	EG = Complete the 4 protocols described
Mackała et al. (35)	POWER breathe®	EG1 = IMT	The intensity was prescribed for the different groups, with different exercise sessions between levels 2–8 of the device, increasing from low to moderate load. The intensity in the RST increased to 75% depending on the capacity of each swimmer	1 s/5 t	4	N/S	EG1 = IMT
							EG2 = IMT + IT
		EG2 = IMT + IT					
		CG = RST					
							CG = RST
Shei et al. (36)	POWER breathe®	EG = 80% MIP	In the EG it increased progressively until the task failed	1 s/3 t	12	N/S	EG = IMT + RST
		PG = Placebo loading IMT					
							PG = IMT Placebo + RST
							CG = RST
		CG = RST					
Troncoso et al. (37)	POWER breathe®	EG = 70% MIP	In the second stage, after the initial measurements, the IMT was incorporated at a load of 70% MIP	2 s/6 t	3	EG = 30 inspiratory efforts 70% MIP	EG = IMT + RST
Bağıran et al. (38)	POWER breathe®	EG = 50% MIP	MIP intensity started at 30% and increased to 50%	2 s/5 t	6	EG = 30 inspiratory efforts 50% MIP	EG = IMT + RST
		CG = RST					CG = RST
Gómez-Albareda et al. (39)	POWER breathe®	EG = 60% MIP up to 80%	EG = increased the load by 10% every 2 weeks to finish the study at an intensity of 80% MIP during his twice-daily sessions	2 s/6 t	6	EG = 3 series of 10 inspiratory efforts 60–80 MIP	EG = IMT 60% up to 80% MIP + RST
		CG = 60% MIP					CG = IMT 60% MIP + RST

EG, experimental group; CG, control group; IMT, inspiratory muscle training; MIP, maximal inspiratory pressure; RMST, respiratory muscle strength training; RMET, respiratory muscle endurance training; RMS, respiratory muscle training; RST, regular swimming training; GT, ground training; HI, high intensity; MI, moderate intensity; IT, individualized training; N/S, not specific.

### 3.3 Evaluation of the effect of respiratory muscle training on performance in swimming athletes

Regarding the evaluation of the effect of IMT on performance in swimming athletes, we found that they used crawl tests at distances of 50, 100, and 200 m (28–30, 34, 35, 37). Kapus (28) showed that he performed two additional tests in butterfly and breaststroked 50 m, Vašíčková et al. (27), who performed apnea test and Gómez-Albareda et al. (39) carry out 3,000 m crawl test. Only 3 studies with measurements pre- and post-IMT found statistically significant changes in test performance (28, 29, 37) (Table 4).

**Table 4:** Evaluation of the effect of IMT on performance, muscle strength and lung function in athlete swimmers.

First author, year	Performance—time trials (s)									Test		
	EG				CG							
	Before		After		Before			After				
Vašíčková et al. (27)	35			50		28.5		45		Apnea		
Kapus (28)	30.89 ± 1.26			30.34 ± 1.78		32.26 ± 2.70		31.72 ± 2.60		Crawl 50 M		
	34.93 ± 2.00			34.17 ± 2.43		35.23 ± 4.81		34.83 ± 4.99		Butterfly 50 M		
	40.57 ± 2.50			39.78 ± 2.64		41.00 ± 2.72		40.23 ± 2.92		Breaststroke 50 M		
	67.53 ± 3.08			66.03 ± 3.23		69.64 ± 7.09		68.10 ± 5.83		Crawl 100 M		
Yañez-Sepulveda et al. (29)	29.4 ± 0.9			28.1 ± 0.8		30.0 ± 1.6		29.9 ± 1.7		Crawl 50 M		
	64.8 ± 2.1			61.9 ± 2.2		69.1 ± 4.5		68.3 ± 4.4		Crawl 100 M		
	144.1 ± 5.2			136.8 ± 5.3		156.4 ± 8		154.4 ± 8.7		Crawl 200 M		
Lomax et al. (30)	Low = 66.6 ± 7.2			Low = 64.5 ± 6.0		Low = 66.2 ± 6.4		Low = 65.7 ± 6.5		Crawl 100 M		
	High = 57.3 ± 3.8			High = 59.2 ± 5.5		High = 61.7 ± 1.0		High = 62.5 ± 5.0				
	Low = 146.9 ± 15.0			Low = 136.7 ± 16.6		Low = 146.0 ± 13.5		Low = 137.3 ± 14.8		Crawl 200 M		
	High = 125.3 ± 9.6			High = 125.1 ± 6.8		High = 126.1 ± 6.6		High = 130.3 ± 7.5				
Troncoso et al. (37)	31.38			32.12		N/E		N/E		Crawl 50 M		
	67.11			68.52		N/E		N/E		Crawl 100 M		
	141.65			142.57		N/E		N/E		Crawl 200 M		
Gómez-Albareda et al. (39)	32:34.50 (01:09.26)			32:17.50 (01:28.49)		33:26.00 (00:49.41)		32:42.25 (01:21.34)		Crawl 3000 M		
First author, year	Muscle strength—maximum inspiratory pressure (PImax) (CmH20)											
	EG						CG					
	Before			After			Before			After		
Vašíčková et al. (27)	−124, 13			−167, 47			−147, 46			−145, 13		
Kapus (28)	−110 ± 21			−173 ± 25			−114 ± 25			−133 ± 11		
Yañez-Sepulveda et al. (29)	−124.8 ± 28.7			−142.8 ± 31.4			−124.5 ± 21.8			−123.7 ± 15.7		
Lomax et al. (30)	Low = 127.4 ± 25.6			Low = 160 ± 25.3			Low = 121.3 ± 24.5			Low = 127.1 ± 28.02		
	High = 114 ± 19.9			High = 168.4 ± 19.08			High = 131.2 ± 23.5			High = 147.3 ± 24		
Ando et al. (31)	−129 ± 6			−163 ± 7			−139 ± 9			−155 ± 7		
Cunha et al. (32)	−73.4			−93,0			−81.2			−99.1		
Ohya et al. (33)	GE (HI) −150			−175			−158			−150		
	GE (MI)-155			−178								
Shei et al. (36)	−108.2 ± 25.0			−205.6 ± 23.8			−114.0 ± 34.2			−119.1 ± 24.8		
Troncoso et al. (37)	−154.2			−175.5			–			–		
Gómez-Albareda et al. (39)	−132.75 (27.42)			−156.75 (21.88)			149.25 (22.82)			171.50 (23.74)		
First author, year	Maximum expiratory pressure (Pemax) (CmH2O)											
	EG						CG					
	Before			After			Before			After		
Vašíčková et al. (27)	99, 48			110, 86			100, 78			99, 63		
Kapus (28)	111 ± 11			129 ± 15			123 ± 20			151 ± 36		
Lomax et al. (30)	135 ± 42			–			133 ± 28			–		
Lung function												
First author, year	Forced vital capacity (FVC) bpm				Forced expiratory volume in one second (FEV1) bpm				FEV1/FVC			
	EG		CG		EG		CG		EG		CG	
	Before	After	Before	After	Before	After	Before	After	Before	After	Before	After
Kapus (28)	–	–	–	–	3.79 ± 0.43	4.09 ± 0.39	3.94 ± 1.11	3.96 ± 0.95	–	–	–	–
Yañez-Sepulveda et al. (29)	5.2 ± 0.7	5.4 ± 0.8	4.7 ± 0.7	4.8 ± 0.7	4.4 ± 0.7	4.6 ± 0.7	4.2 ± 0.6	4.2 ± 0.6	84.6 ± 7.7	84.0 ± 7.3	88.8 ± 5	88.0 ± 4.6
Lomax et al. (30)	5.23 ± 1.22	–	4.48 ± 1.25	–	4.40 ± 1.14	–	3.94 ± 1.08	N/E	84 ± 8	–	88 ± 6	–
Cunha et al. (32)	5	5.1	4.3	4.3	4.4	4.5	–	3.7	–	–	–	–
Ohya et al. (33)	HI = 5.79	HI = 5.70	5.76	5.71	–	–	–	–	–	–	–	–
	MI = 5.92	MI = 5.81			–	–			–	–		
Wilson et al. (34)	5.90 ± 0.95	–	–	–	4.84 ± 0.81	–	–	–	–	–	–	–
Mackała et al. (35)	4.23 ± 0.24	4.44 ± 0.28	4.22 ± 0.27	4.25 ± 0.26	3.36 ± 0.16	3.90 ± 0.22	3.45 ± 0.23	3.47 ± 0.23	79.49 ± 2.67	79.820 ± 3.74	81.82 ± 3.24	81.93 ± 3.05
Shei et al. (36)	5.02 ± 0.24	4.93 ± 0.27	4.94 ± 0.28	5.04 ± 0.10	4.21 ± 0.42	4.13 ± 0.34	4.31 ± 0.21	4.24 ± 0.45	–	–	–	–
Bağıran et al. (38)	4.23 ± 1.52	4.91 ± 1.12	4.29 ± 1.52	4.32 ± 1.12	3.52 ± 0.97	3.69 ± 1.32	3.52 ± 0.97	3.69 ± 1.32	90.51 ± 2.12	92.81 ± 3.27	89.37 ± 2.16	90.11 ± 1.47

EG, experimental group; CG, control group; IMT, inspiratory muscle training; HI, high intensity; MI, moderate intensity; M, meters.

### 3.4 Evaluation of the effect of respiratory muscle training on strength in swimming athletes

In the experimental group the baseline values of MIP ranged from −73.4 CmH2O (28) to −154.2 CmH2O (37), after IMT, the values ranged from −93.0 CmH2O (32) to −205.6 ± 23.80 CmH2O (37). For the control group were reported baseline values between −81.2 CmH2O (32) and −149.25 CmH2O (39), and post-IMT the range was between −99.1 CmH2O (29) and −171.5 CmH2O (39). Three studies did not report results on MIP (34, 35, 38).

Only three studies reported MEP values (27, 28, 30). In the IMT group, initial values were reported from 99.48 CmH2O (27) to 135 ± 42 CmH2O (30) and post-IMT 110.86 CmH2O (27) to 129 ± 15 CmH2O (28). One study did not report final values after training (30). While in the control group, basal values ranged from 100.78 CmH2O (27) to 133 ± 28 CmH2O (30) and subsequent values ranged from 99.63 CmH2O (27) to 151 ± 36 CmH2O (28). Ten articles (29, 31–39) did not report MEP results (Table 4).

### 3.5 Evaluation of respiratory muscle training on pulmonary function in swimming athletes

Table 4 shows the results of FVC, FEV1, and FEV1/FVC ratios. The FVC baseline in the IMT group had a range of 4.23 ± 0.24 L (35) to 5.92 L (33), and post-IMT a range of 4.44 ± 0.28 L (35) to 5.81 L (33). In the control group, the initial values ranged from 4.22 ± 0.27 L (35) to 4.94 ± 0.28 L (36) and later from 4.25 ± 0.26 L (35) to 5.04 ± 0.10 L (36). One study reported no subsequent values (30) and 6 studies reported no baseline or subsequent values (27, 28, 31, 34, 37, 39).

The FEV1 results for the IMT group showed baseline values ranging from 3.36 ± 0.16 L (35) to 4.84 ± 0.81 L (33). On the other hand, the control group had baseline values ranging from 3.45 ± 0.23 L (35) to 4.31 ± 0.21 L (36), and post-measurement values ranging from 3.47 ± 0.23 L (35) to 4.24 ± 0.45 L (36). Post-intentions values were not reported in two studies (30, 34) while five studies did not report either baseline or post-values (27, 31, 33, 37, 39).

The FEV1/FVC ratio in the IMT group had a baseline range of 79.49 ± 2.67 L (27) to 90.51 ± 2.12 L (38), and post-IMT values ranged from 79.820 ± 3.74 L (35) to 92.81 ± 3.27 L (38). Meanwhile, the control group had a baseline range of 81.82 ± 3.24 L (35) to 89.37 ± 2.16 L (38), and post-measurement values ranged from 81.93 ± 3.05 L (35) to 90.11 ± 1.47 L (38). However, it is worth noting that nine studies did not report these results (27, 28, 31, 32, 36, 37, 39).

### 3.6 Quality of the studies

The mean PEDro score for the CCTs described in Table 5 was High for two studies (27–32) and Intermediate for 9 studies (28–33, 35, 36, 38, 39). The Minor mean score for the PLS for these two studies was 13 and 17, respectively (Table 5), where the ideal score would be 16 for non-comparative studies. The most frequent omissions in the study design or its reporting were the following: the randomization process was not concealed and the non-blinding of subjects and evaluators.

**Table 5:** Methodological quality of the studies controlled clinical trials (PEDro score) and prospective longitudinal observational studies (minors scale).

Methodological quality of the studies controlled clinical trials (PEDro score)													
First author, year	1^a^	2	3	4	5	6	7	8	9	10	11	Total	Methodological quality
Vašíčková et al. (27)	–	1	1	1	1	1	1	1	1	1	1	10	High
Kapus (28)	–	1	0	1	0	0	0	1	1	1	1	6	Intermediate
Yañez-Sepulveda et al. (29)	–	1	0	1	0	0	0	1	1	1	1	6	Intermediate
Lomax et al. (30)	–	1	0	1	0	0	0	1	1	1	1	6	Intermediate
Ando et al. (31)	–	1	0	1	0	0	0	1	1	1	1	6	Intermediate
Cunha et al. (32)	–	1	0	1	0	1	1	1	1	1	1	8	High
Ohya et al. (33)	–	1	0	1	0	0	0	1	1	1	1	6	Intermediate
Mackała et al. (35)	–	1	0	1	0	0	0	1	1	1	1	6	Intermediate
Shei et al. (36)	–	1	0	1	0	0	0	1	1	1	1	6	Intermediate
Bağıran et al. (38)	–	1	0	1	0	0	0	1	1	1	1	6	Intermediate
Gómez-Albareda et al. (39)	–	1	0	1	0	0	0	1	1	1	1	6	Intermediate
Methodological quality of prospective longitudinal observational studies minors scale													
First author, year	1	2	3	4	5	6	7	8	9	10	11	12	Total
Wilson et al. (34)	2	1	2	2	2	2	2	2	0	0	0	2	17
Troncoso et al. (37)	2	1	2	2	0	1	2	1	0	0	0	2	13

PEDro scale criteria: (1) choice criteria were specified (^a^- this item is not used to calculate the PEDro score), (2) subjects were randomly assigned to groups (in a crossover study, subjects were randomized as they received treatments), (3) allocation was concealed, (4) groups were similar at baseline with respect to major prognostic indicators, (5) all subjects were blinded, (6) all therapists who administered the therapy were blinded, (7) all raters who measured at least one key outcome were blinded, (8) measurements of at least one of the key outcomes were obtained from more than 85% of subjects initially assigned to groups, (9) results were presented for all subjects who received treatment or were assigned to the control group, or where this could not be, data for at least one key outcome were analyzed by “intention to treat”, (10) the results of statistical comparisons between groups were reported for at least one key outcome, (11) the study provides point and variability measures for at least one key outcome. 1 = item met, 0 = item not met. Quality criteria: ≥7 high quality, 5–6 intermediate quality, ≤4 low quality.

Minors scale criteria: (1) clearly defined objective, (2) inclusion of patients consecutively, (3) prospective data collection, (4) results appropriate for the study objective according to the intention to treat, (5) unbiased outcome assessment (blinding), (6) follow-up period appropriate for study objective, (7) loss to follow-up less than 5%, (8) calculation of study sample size, 95% confidence interval, (9) an adequate control group, (10) groups managed at the same time both control and study, (11) baseline equivalence of groups, (12) adequate statistical analysis. 0 = not reported, 1 = reported but inadequate, 2 = reported and adequate. The ideal score would be 16 for non-comparative studies and 24 for comparative studies.

### 3.7 Association of IMT with MIP, FEV1, and FVC

**Figure 2:**
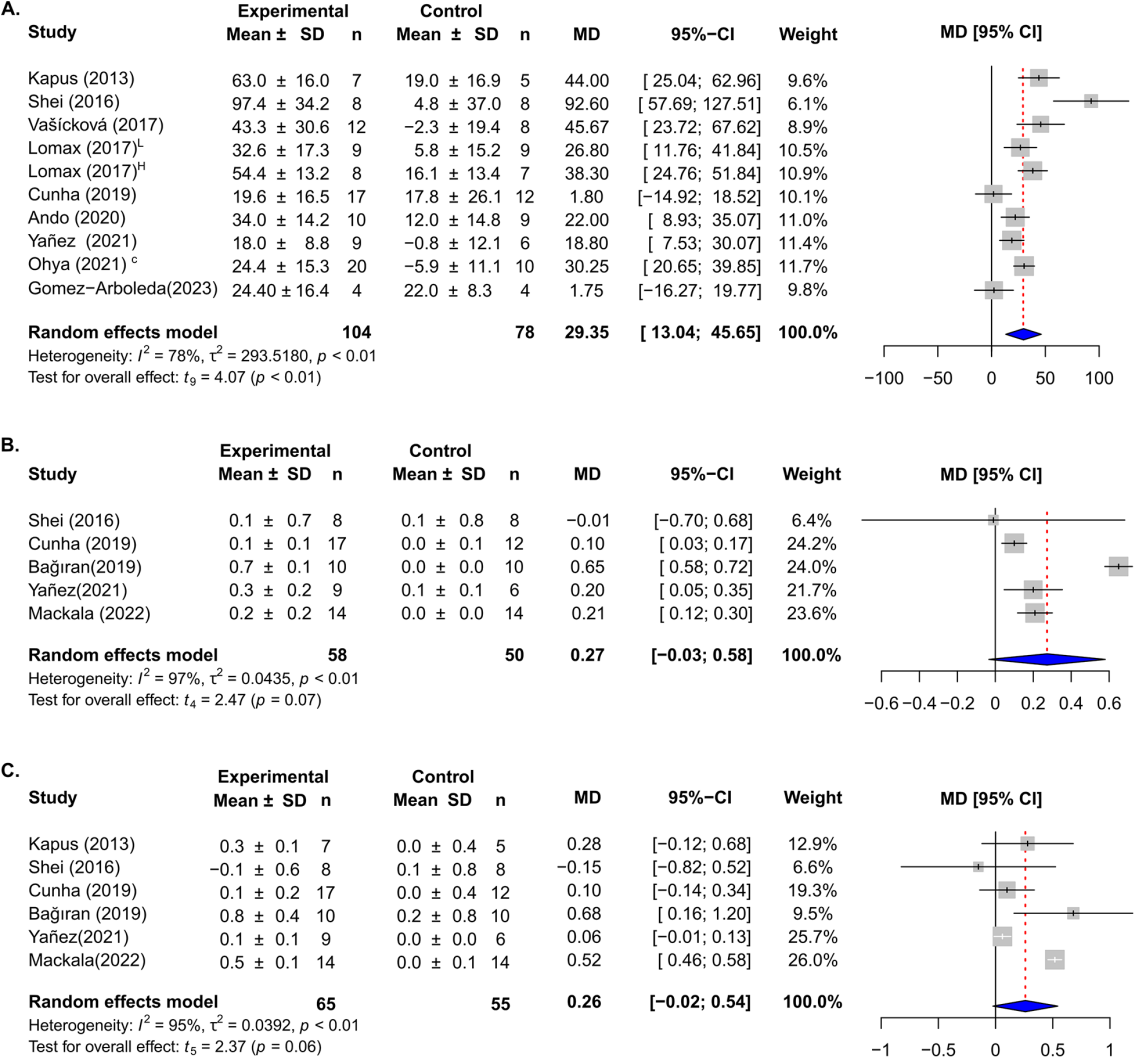
Forest plots of the effect of IMT on maximal inspiratory pressure **(A)**; forced expiratory volume **(B)** and forced vital capacity **(C)**. Horizontal lines indicate confidence intervals for each study. Horizontal diamonds of blue color show overall confidence intervals and the midline in red color indicates the mean difference (MD).

For the quantitative synthesis of MIP, we selected 9 articles (28–33, 35, 36, 38, 39) (IMT group *n* = 104 and control group *n* = 78). The results showed that IMT in swimmers increased the MIP 29.35 cmH2O (CI-95%: 13.04–45.65 cmH2O, *p* < 0.01), the heterogeneity of this result was high (*I*2 = 78%) (Figure 2A).

The effect of IMT on FEV1 was assessed in six studies (28, 29, 32, 35, 36, 38) (IMT group *n* = 65 and control group *n* = 55), the analysis showed that the intervention increased by 0.26 L, but did not reach to be significant and showed a substantial heterogeneity (CI-95%: −0.02 to 0.54, *p* = 0.06, *I*2 = 95%) (Figure 2B).

For FVC we pooled five studies (27, 29, 30, 37, 38) (IMT group = 58 and control group *n* = 50), and quantitative synthesis showed a not significant increase of 0.27 L (CI-95%: −0.03 to 0.58, *p* = 0.07) with high variation (*I*2 = 97%) (Figure 2C).

### 3.8 Sensitivity and meta-regression analysis

The meta-analysis showed a high heterogeneity (*I*2 > 75%) in all the outcomes studied. The sensitivity analysis suggests that the results for MIP are robust, and the removal of studies did not affect considerably the effect and the heterogeneity of the meta-analysis (Supplementary Material 1). In the case of FEV1, the omission of Mackala et al. (35) reduced the heterogeneity (*I*2 = 42%) but without a significant effect (MD = 0.08 L, CI-95%: −0.05 L to 0.20 L, *p* = 0.16) (Supplementary Material 1). The sensitivity analysis for FVC after removing the study of Bağiran et al. (38) showed a significant effect (MD = 0.15l, CI-95%: 0.05–0.25l, *p* = 0.02) and a substantial reduction of the heterogeneity (*I*2 = 31%) (Supplementary Material 1). We performed a subgroup analysis considering the type of control group; control group with IMT sham or control group with regular swimming training. We found that the pooled effect for MIP in studies with an IMT sham group was not significant (MD = 16.35, CI-95%: −14.32 to 47.02, *I*2 = 78%) (Supplementary Material 2), while for FEV we found a significant effect in the studies with a control group with regular swimming training (MD = 0.52, CI-95%: 0.33–0.71, *I*2 = 52%) (Supplementary Material 3).

Meta-regression analyses for potential factors related to the effect of IMT on MIP revealed no significant relationship with MIP basal (*P* = 0.88), age (*P* = 0.90), and duration of intervention (*P* = 0.76) (Supplementary Material 4).

### 3.9 Publication bias

The LFK index showed minor asymmetry for MIP (LFK = 1.26), no asymmetry for FEV (LFK = −0.92), and FVC (LFK = −0.93) (Supplementary Material 5), indicating no obvious publication bias.

## 4 Discussion

This systematic review and meta-analysis found that training with IMT in elite and non-elite swimmers significantly increased MIP, this effect was not related to age, duration of intervention, or basal MIP of the participants. We also found that IMT was not associated with changes in MEP, FEV1, and FVC.

Schoenfeld et al. (40), suggested that different training regimens can maximize performance or hypertrophy in athletes. Evidence shows that different exercise volumes, intensity, and timing, induce different degrees of hypertrophy and muscular strength (41). In our study, we found a significant increase in the MIP post-IMT, when training was carried out with intensity progression. This finding is similar to those reported by Kilding et al. (8) (a study not included in the meta-analysis because its publication date was outside the inclusion criteria) who evaluated the effect of IMT on the performance in the 100 and 200-m free events and found an increase in both the performance and the strength of the inspiratory muscles in swimmers. Respiratory muscle training is linked to enhanced endurance performance in intermittent incremental tests, constant load tests, and time trials. It also boosts respiratory muscle endurance and strength, decreases the perceived exertion or breathlessness, and lessens respiratory fatigue during exercise in hypoxic conditions (42).

The increase in respiratory muscle strength can improve the ventilatory function of the rib cage, allowing for greater and faster thoracic excursion. It is well known that there is greater lung ventilation to higher the thoracic excursion. The thoracic wall excursion is determined by several factors, such as compliance of soft tissue structures surrounding the thorax, chest form, and respiratory muscle strength (43). The most important component of the increase in lung volume is the rise in the chest cavity's rostral-caudal diameter and the anterior-posterior and transverse diameter, as a result of the action of the diaphragm and the external and internal intercostal muscles respectively (5). The increase in lung volume has two direct effects on the alveolar-capillary membrane: it increases the area and decreases the thickness of the alveolar-capillary membrane, both processes favoring gas diffusion capacity. Stronger inspiratory muscles, reflected by increased MIP, can influence the athlete's performance associated with increased gas exchange and tissue oxygen bioavailability (44).

Although the increase in the strength of the respiratory muscles is associated with an increase in lung volumes and capacities (45), this was not reflected in our study. We did not find an increase in pulmonary function assessed by FVC and FEV1 after IMT. The fact that training mainly affects the respiratory musculature and not the resistance and distensibility of the air conduction zones of the lung could explain the no effect on the FVC and FEV, despite exerting more power and having a higher thoracic excursion (46). However, other studies in other sports disciplines have shown significant differences in lung volumes and capacities after IMT. Vasconcelos et al. (47), on basketball athletes, using a Threshold IMT® device, 5 times a week, for 4 weeks, 30 repetitions at 50% of the MIP, reported a significant change in FEV1, FVC, and peak expiratory flow. Koç et al. (48), in taekwondo athletes, showed significant changes in FVC, slow FVC, and maximal voluntary ventilation. On the other hand, during the research process of the systematic review, other forms of IMT were found, such as the use of a Respiratory Dead Space Addition Device (ARDS®). Szczepan et al. (49) used ARDS in recreational swimmers, for 6 weeks, twice a week, for 50 min in freestyle, however, the study reports no differences in muscle strength or spirometric parameters, and only one of the groups showed an increase in peak tidal volume.

We found that the protocols of IMT in swimmers included durations between 3 and 12 weeks, 1–2 sessions per day, 3–6 times per week, and 10–30 dynamic respiratory efforts, starting with 50% of MIP with progression up to 80% and the most common IMT device used was the Power Breathe®. This protocol is similar to reported in other studies as the Kilding et al. (8), in which the group of swimmers performed training for 6 weeks, 2 times a day, using Power Breathe®, however, the progression of MIP was not described. De Asís-Fernández et al. (50), applied IMT in divers, 3 times a week for 4 weeks, once a day, with a PowerBreathe® device. We found that protocols of IMT sessions ranged from 10 to 30 dynamic inspiratory efforts, with an initial pressure threshold load in most studies at 50% of MIP, and a progression of intensity between 70% and 80%. Studies in other sports show similar patterns in starting intensity and progression. Cavalcante-Silva et al. (51) on soccer players used a protocol of 15–30 dynamic respiratory efforts, starting at 50% of the MIP, although the progression was not specified. In runners, Rozek-Piechura et al. (52) used a protocol of 30 repetitions, starting with 50% of the MIP and progressing to 60% of the MIP between weeks 4 and 6 and, reaching 70% in weeks 7 and 8. The above suggests that the IMT protocols in elite, and non-elite swimmers are very similar to those used in athletes’ aquatic and land sports.

Our study had limitations such as not all included studies measured simultaneously performance, strength and pulmonary function tests. In addition, some studies did not perform measurements before and after the IMT intervention, which made comparative analysis challenging. A second difficulty was the classification of elite and non-elite athletes, due to different ways of reporting details about the athlete's condition. Besides. some studies could not be included because used IMT devices other than Threshold IMT®, or PowerBreathe®, such as normocapnic hyperpnea or ARDS®, or because were performed in swimmers with some disabilities (53).

The results of this meta-analysis can be valuable to designing training protocols for the respiratory muscles with IMT, especially in swimming to improve respiratory function in athletes. However, further studies are required to evaluate the effects of different IMT modalities, lung volumes, muscle strength, and their impact on technique and athletic performance.

## 5 Conclusions

Incorporating IMT in the training of elite and non-elite swimmers contributes to improvements in muscle strength, particularly MIP. This effect can be achieved by practicing IMT for 3–12 weeks, with 1–2 daily sessions, 3–6 times per week, performing 30 repetitions, and starting at 50% of MIP and gradually progressing up to 80% of MIP.

## Data Availability

The original contributions presented in the study are included in the article/Supplementary Material, further inquiries can be directed to the corresponding author.
