# Supplementary material for: Effects of inspiratory muscle training on lung function parameter in swimmers: a systematic review and meta-analysis
Source: Front Sports Act Living. 2024 Sep 16;6:1429902. doi: 10.3389/fspor.2024.1429902 (PMC11439704; doi:10.3389/fspor.2024.1429902)
Supplement: Supplementary file 1 [file Table1.docx]

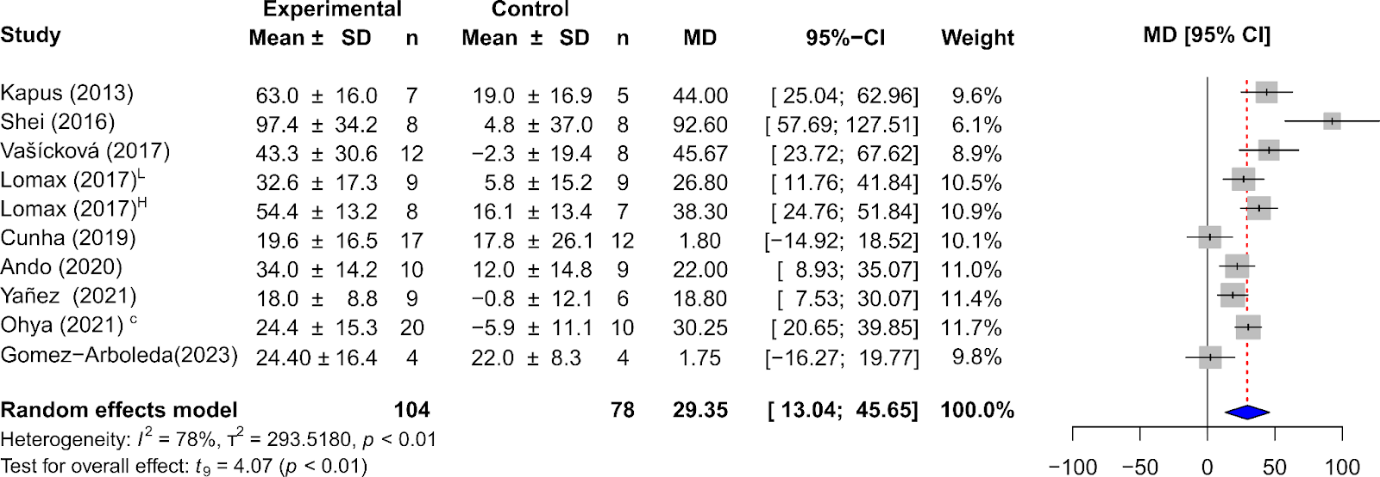


**Supplementary material 1.** Forest plots of the effect of IMT over Maximal Inspiratory Pressure (MIP). Horizontal lines indicate confidence intervals for each study. Horizontal diamonds of blue colour show overall confidence intervals and the midline in red colour indicates the mean difference (MD).
