# Supplementary figures and images for: Effects of inspiratory muscle training on lung function parameter in swimmers: a systematic review and meta-analysis
Source: Front Sports Act Living. 2024 Sep 16;6:1429902. doi: 10.3389/fspor.2024.1429902 (PMC11439704; doi:10.3389/fspor.2024.1429902)

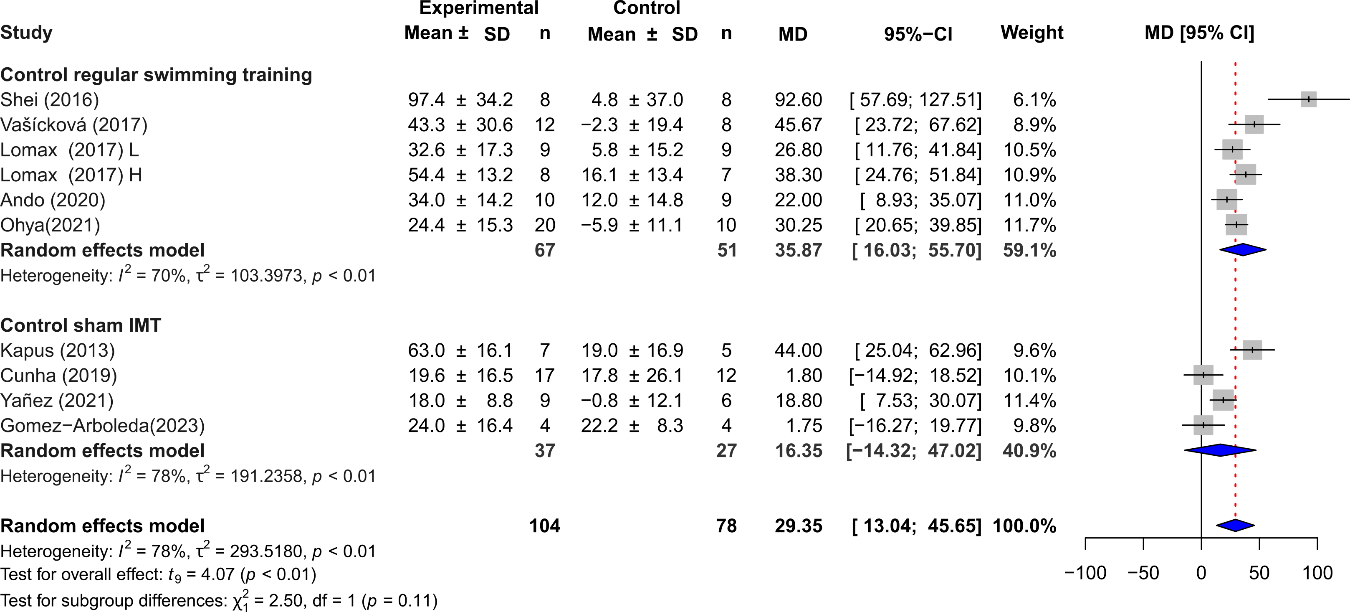


Supplementary material 2. Subgroup analysis of MIP according to the type of control group.

Supplement: Supplementary file 2 [file Table2.docx]
