# Supplementary material for: Effects of inspiratory muscle training on lung function parameter in swimmers: a systematic review and meta-analysis
Source: Front Sports Act Living. 2024 Sep 16;6:1429902. doi: 10.3389/fspor.2024.1429902 (PMC11439704; doi:10.3389/fspor.2024.1429902)
Supplement: Supplementary file 3 [file Table3.docx]

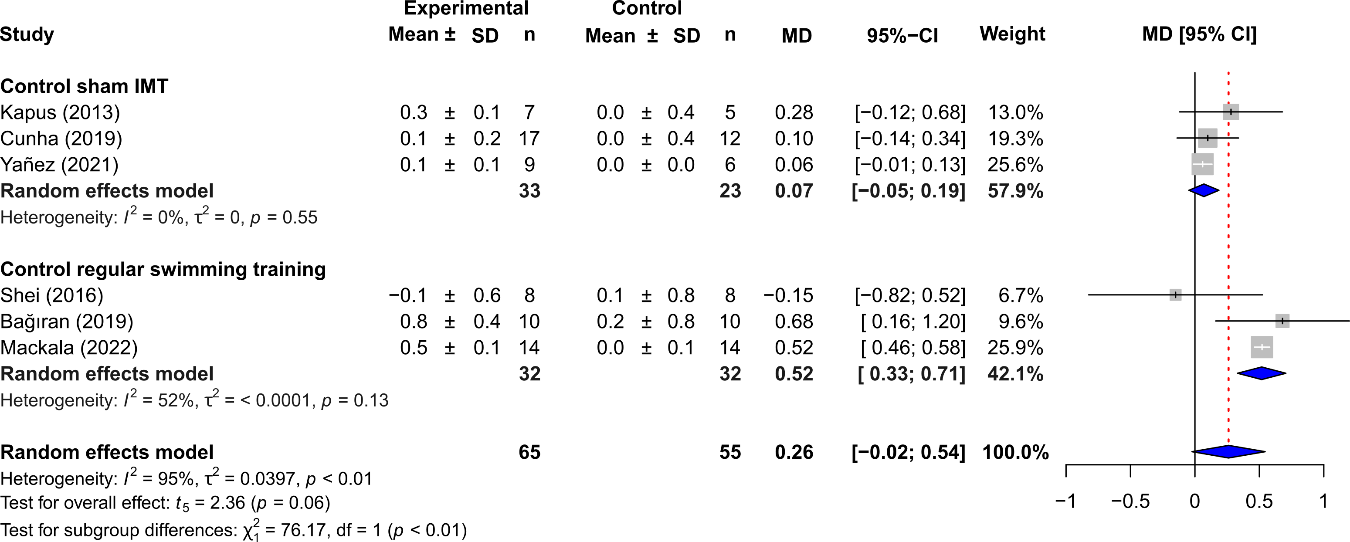


Supplementary material 3A. Subgroup analysis of FEV according to the type of control group.


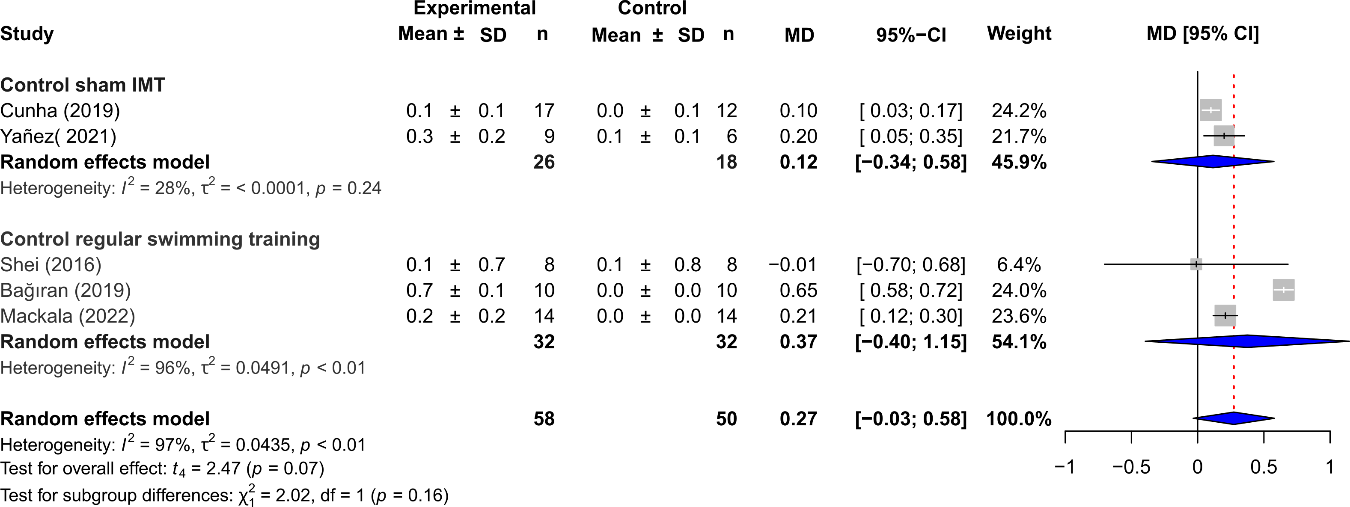


Supplementary material 3B. Subgroup analysis of FVC according to the type of control group.
