# Supplementary material for: Effects of inspiratory muscle training on lung function parameter in swimmers: a systematic review and meta-analysis
Source: Front Sports Act Living. 2024 Sep 16;6:1429902. doi: 10.3389/fspor.2024.1429902 (PMC11439704; doi:10.3389/fspor.2024.1429902)
Supplement: Supplementary file 4 [file Table4.docx]

Supplementary material 4. Analysis of meta-regression for mean differences against potential effect moderators of MIP.

|  | **SE** | **P-value** | **95% CI Lower** | **95% CI Lower** |
| --- | --- | --- | --- | --- |
| **MIP basal** | 0.0801 | 0.8800 | -1.1291 | 1.2893 |
| **Age** | -0.3722 | 0.9043 | -7.2900 | 6.5456 |
| **Duration of intervention** | 0.956 | 0.7601 | -6.0205 | 7.9327 |
