# Supplementary material for: Effects of inspiratory muscle training on lung function parameter in swimmers: a systematic review and meta-analysis
Source: Front Sports Act Living. 2024 Sep 16;6:1429902. doi: 10.3389/fspor.2024.1429902 (PMC11439704; doi:10.3389/fspor.2024.1429902)
Supplement: Supplementary file 5 [file Table5.docx]

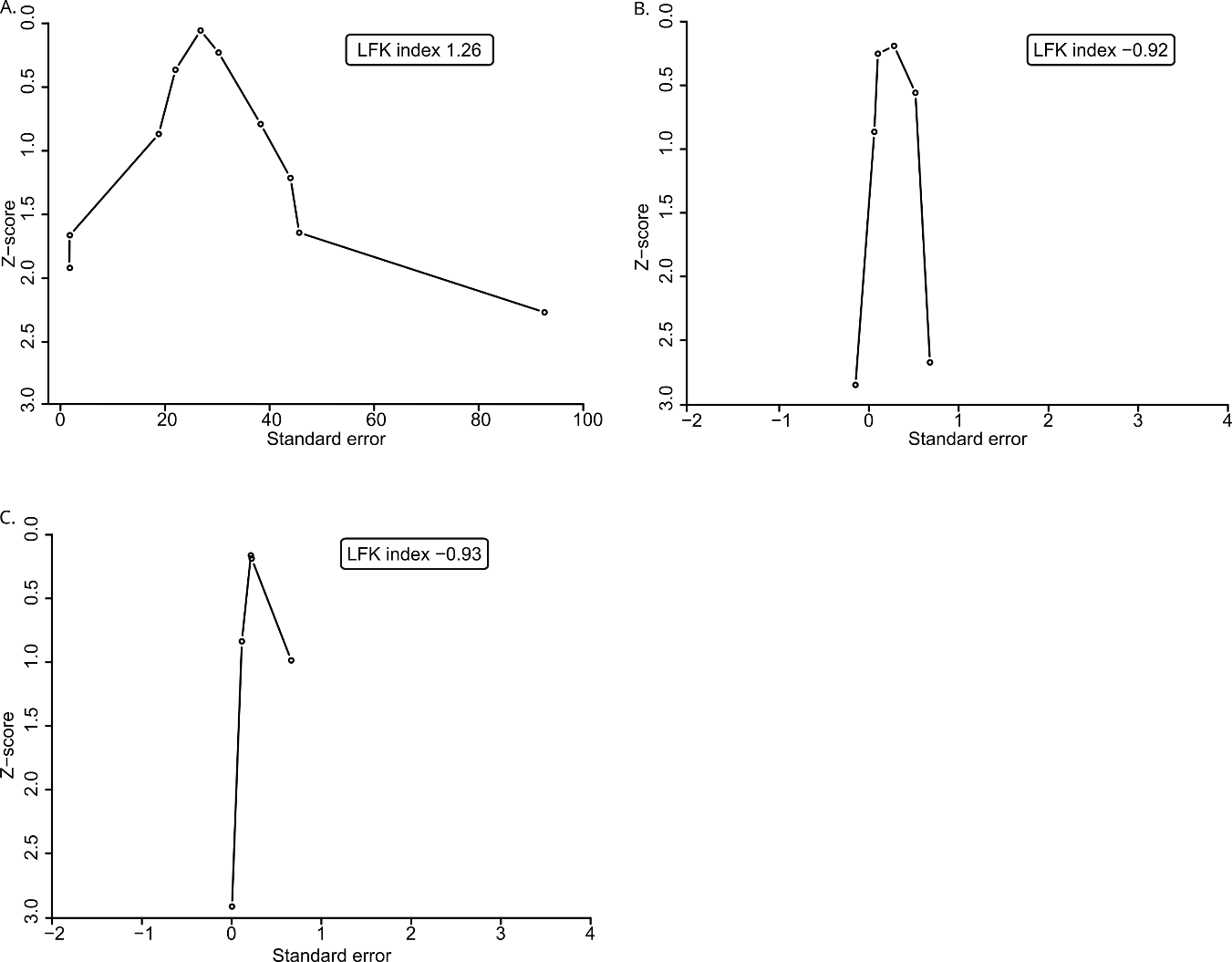


Supplementary material 5. Doi plot and asymmetric index (LFK) for risk of publication bias of MIP (Figure 1A), FEV (Figure 2B), and FVC (Figure 2C).
